# Sociodemographic and Healthcare Factors Associated with Stunting in Children Aged 6–59 Months in the Urban Area of Bali Province, Indonesia 2018

**DOI:** 10.3390/nu15020389

**Published:** 2023-01-12

**Authors:** Anak Agung Ngurah Kusumajaya, Rofingatul Mubasyiroh, Sudikno Sudikno, Olwin Nainggolan, Hertog Nursanyoto, Ni Ketut Sutiari, Kadek Tresna Adhi, I Made Suarjana, Pande Putu Januraga

**Affiliations:** 1 Health Polytechnic, Ministry of Health, Denpasar 80224, Indonesia; 2 Research Center for Public Health and Nutrition, National Research and Innovation Agency, Jakarta 10340, Indonesia; 3 Health Development Policy Agency, Ministry of Health, Jakarta 10560, Indonesia; 4 Department of Public Health and Preventive Medicine, Medical Faculty, Udayana University, Denpasar 80361, Indonesia

**Keywords:** stunting, education level, extended family, iron tablets, Bali, Indonesia

## Abstract

Stunting is a worldwide public health concern, including in Indonesia. Even when living in an urban area with urban characteristics, it is still possible for children to be at risk of stunting. The aim of this study was to determine the sociodemographic and healthcare factors associated with stunting in a province experiencing tourism growth, namely, Bali. Cross-sectional data on Bali Province from the Indonesian Basic Health Research Survey (Riskesdas, 2018) were used as the basis for the research analysis. A total of 846 respondents under five years of age were analyzed, indicating a stunting prevalence of 19.0%. Multivariate logistic regression demonstrated low maternal educational attainment (adjustedOR = 1.92; 95% Confidence Interval = 1.24–2.97), the inadequate consumption of iron tablets during pregnancy (adjustedOR = 1.56; 95% Confidence Interval = 1.08–2.24), and no extended family (adjustedOR = 1.55; 95% Confidence Interval = 1.07–2.26) as being significantly associated with stunting. According to these findings, sociodemographic and healthcare factors are associated with stunting in urban Bali. Improving women’s education, ensuring sufficient iron tablets are consumed during pregnancy, and encouraging the involvement of the extended family in childcare are recommended.

## 1. Introduction

Stunting is a global public health issue. According to United Nations Children’s Fund (UNICEF), the World Health Organization (WHO), and the World Bank’s 2018 global report, approximately 150.8 million children under the age of five are stunted, or 22.2% of all children under the age of five. Of these children, 83.6 million are spread across Asia, and specifically, 25.7% are from Southeast Asian countries [1]. Stunting refers to the condition when a child has impaired growth and development for his or her age as a result of malnutrition. Factors contributing to stunting may be found during pregnancy or the early stages of childhood growth [2]. Even though progress has been made in terms of lowering child mortality, Indonesia still needs to address the issue of stunting [3]. The prevalence of stunting dropped in Indonesia from 37.2% in 2013 to 29.9% in 2018 [4]. Even though it has decreased, its stunting rates are among the highest in the world, higher than most regional and income-level peers and on par with fragile sub-Saharan African countries such as Mali, Ethiopia, and Djibouti [3]. Based on the Sixty-Fifth World Health Assembly, each country must achieve a 40% reduction in stunting rates between 2012 and 2025, or a 3.9% reduction per year. This means that Indonesia still has a duty to pursue the achievements of 2025 [2,5].

When compared to normal children, children with stunting have both short- and long-term consequences, including irreparable brain damage, stunted growth, adult vulnerability to chronic diseases, reduced educational performance, and a tendency to earn lower wages [6,7]. The Indonesian government remains dedicated to stunting mitigation, as specified in the Medium-Term Development Plan, both in 2015–2019 and 2020–2024, given the high rate of stunting and its consequences [8,9]. Their plans were then carried out across agencies as part of the national strategy’s objective to combat stunting through nutrition-specific (supplementary feeding for pregnant mothers, exclusive breastfeeding counseling, prenatal health checks, supplementation for children, complete immunization, and growth monitoring and promotion programs) and sensitive interventions (access to water and sanitation, access social insurance, awareness, behavior change, parenting, and caring practices, and access to nutritious food programs) and intervention convergence (about leadership, coordination, technical assistance, community empowerment, and social accountability [3].

Previous research, both globally and in Indonesia, has found numerous factors related to stunting, including individual, family, and community factors. Individual characteristics include gender, birth weight and length, morbidity, infection, exclusive breastfeeding, and childhood diet. Family factors consist of maternal height, the mother’s education, family food availability, type of family, and economic status. Meanwhile, access to clean water and sanitation, culture, access to healthcare, and type of residence are all examples of characteristics of the household environment [10,11]. Our study presents factors that may enrich and sharpen the national strategy for tackling stunting in Indonesia, especially in Bali Province.

Indonesia has notable differences in the rate of stunting across its regions. Bali was one of the provinces with a decreased number of stunting cases in 2021 [12]. The rates of stunting dropped dramatically in Bali from 14.40% (2019) to 10.90% (2021). Despite the lower number of stunting cases, closer examination reveals the significant differences in stunting cases between urban and rural areas, as well as between districts and cities in Bali Province. The expansion of Bali’s tourism industry has prompted a number of locations to develop into commercial areas, giving the city a high per capita income and rapid economic growth [13]. Bali Province is the province with the best stunting profile in Indonesia. With its economic potential, Bali wants to create a stunting-free Bali. With the nearest target of 2024, the stunting rate has decreased to 6.15% [14]. Knowing the factors that are still related to stunting in Bali, they can be input for intervention steps to accelerate a stunting-free Bali. In this context, this study aimed to determine associations between sociodemographic and healthcare factors and the prevalence of stunting in urban Bali Province.

## 2. Materials and Methods

### 2.1. Study Design

This study was a cross-sectional analysis using secondary data collected from the Indonesian National Health Survey (Riskesdas) in 2018. Riskesdas is a national survey that considers representatives of all households across 34 provinces and 514 districts, with cities of Indonesia as the study population. Using a multistage systematic random sampling method, the household level was the smallest sample unit chosen for this study. The first stage selected groups of census blocks and classified them as key sampling units (PSUs). The second step employed a probability proportional to the enrolment size design to identify a census block from each PSU. The master frame of 720,000 census blocks (CB) collected in the 2010 Indonesian population survey by the Indonesian Central Bureau of Statistics or Badan Pusat Statistik (BPS) was selected as the sampling frame using the probability proportional to size (PPS) approach. Another PPS that looked at urban–rural distribution used linear systematic sampling: implicit stratification at the 30,000 CB level, followed by implicit stratification at the household level. The third stage involved the systematic random sampling of ten census buildings from each census block [15]. Data were gathered on the risk factors and indicators of health status for each family member.

Bali Province is one of the provinces of Indonesia and is known as the “Island of the Gods.” The 2018 Riskesdas survey in Bali included 576 census blocks spread throughout nine regencies or cities, with 1470 children under the age of five being successfully interviewed [4]. This study only looked at urban-related household data from Bali Province. The status of an administrative area at the village or kelurahan level that fits the requirements for a designated urban area is known as “urban.” An area is considered urban if the indicator scores for population density, the percentage of agricultural households, and ownership of or access to urban facilities (schools, markets, stores, movies, hospitals, hotels, houses with electricity, houses with telephones) totals 10 [16]. The data subset was obtained upon a request that was authorized by the Health Development Policy Agency of the Indonesian Ministry of Health.

### 2.2. Sample and Data Collection

Data on male and female children under the age of five years were included in the research samples gathered as part of the 2018 Basic Health Research Survey. The information consisted of demographic characteristics, medical conditions, health care used, and health behaviors. Face-to-face interviews with respondents were conducted by certified enumerators with a working background in the health sector. They made home visits to respondents to collect data on the respondents’ weights and heights, as well as their overall health. They used a paper questionnaire, which was afterward input into a computer. A district/city-level field supervisor oversaw the data collection procedures to assure the accuracy of the data.

Riskesdas collected anthropometric information for all children in the household. However, in our analysis, the variables of health services accessed by mothers during pregnancy, namely, antenatal care (ANC), desire for pregnancy, and use of contraception, were only answered by mothers for the condition of the last child in the household, so our analysis only included data on the youngest child in the household.

All respondents, across all age groups and sexes, had their height measured. Two enumerators worked together to complete this measurement process; one performed the actual measurement, while the other recorded the results. Height measurements also included recording whether the children were standing or recumbent. With a 0.1 cm precision, the height measurement tool had a maximum measurement of up to 2 m. For children who could support themselves, they were asked to stand on bare feet, cover their heads, and move their backs, buttocks, and calves to the measurement device as closely as possible. For children who could not or did not want to stand on their own, after removing footwear, head coverings, and pampers, the head was moved closer to the measuring instrument board to ensure the imaginary line (the point of the ear lobe to the tip of the eye) was perpendicular to the floor where the children lay.

The number of children 6–59 months analyzed in urban areas of Bali was 768. Using the formula of the Lemeshow health study sample for two-sample hypothesis testing of proportions [17], with an estimated prevalence of stunting in urban areas from previous studies (using Tanzania results) of 25.6% [18] and a 95% confidence interval 0.10 wide (0.05 on either side), the minimum sample required was 293 children under five. Thus, the number of samples we analyzed was larger than the minimum needed.

### 2.3. Outcome Variable

The research unit of analysis was children under the age of five years, along with their weight and height measurement results. The primary research variable was stunting. Stunting is defined as a measure of children’s nutritional status based on their height, according to the 2005 child growth standard of the World Health Organization and guidelines of the Indonesian Ministry of Health [19,20]. Stunting is also defined as a height–forage (HAZ) less than minus 2 standard deviations (−2 SD), according to the child growth standard median from the WHO Multi-Center Growth Reference Study. To calculate the percentage of stunted children under five years old, HAZ scores were recorded. According to the WHO reference population, children whose HAZ scores fell between −2 SD and −6 SD were coded 1, and children whose HAZ scores fell between −2 SD and +6 SD were coded 0.

### 2.4. Sociodemographic Variables

Sociodemographic variables in this study included the mother’s education, mother’s employment status, the mother’s emotional mental disorder, mother’s age at delivery, mother’s desire for pregnancy, child’s sex, economic status, presence of extended family, types of water sources used in the household, social security, receipt of direct cash assistance/expected family programs, and ownership of a toilet in the household.

The mother’s education level was in the high category if she had completed a minimum of senior high school education and in the low category if she had received and completed junior high school or lower education. A mother’s employment status was categorized as working if she had a job. If she did not have a job or was still a student, she was categorized as not working. We calculated the age of the mother at delivery in completed years based on the family card information regarding the birth dates of the mother and the child. The mother’s age at delivery was divided into two categories: non-risk delivery for the age range of >=20 to <=35 completed years and risk delivery for the age range of <20 or >35 completed years [21]. The desire to become pregnant was divided into two categories: desired or undesired. The sex of the children was categorized into two categories: male and female.

Mental emotional disorder status was measured using the Self Reporting Questionnaire-20 (SRQ-20) instrument. The SRQ-20 is a screening instrument for common mental disorders that have been validated for internal validity with a high Cronbach score (>0.80) [22]. In addition, the SRQ-20 has been translated and validated in Indonesian [23]. Every question had a yes/no response. If the answer was “yes,” it was given a score of “1”, and “no” was given a score of “0”. All answers were added together. Respondents with a score of 6 or higher were classified as having mental disorders [23].

A household’s economic status was determined by the quintile of the index of ownership. The index of ownership was calculated using household ownership characteristics such as the ownership of a house or building, the type of wall, roof, and floor in the residence, the type of water source and toilet facilities, and the type of electric provision. The index of ownership as economic status was constructed using principal component analysis, which is divided into 5 quintiles. The household was considered not poor if it fell into quintile categories 3, 4, or 5 (three top wealth quintiles: middle, richer, and richest), and it was considered poor if it fell into quintile categories 1 and 2 (bottom two wealth quintiles: poorest and poorer). This was classified similarly to several previous studies [24,25]. A family with other adults (17 years old and above) relatives or members living at the house—large families with blood relatives—were referred to as an extended family [26,27].

### 2.5. Healthcare Variables

Healthcare variables consist of antenatal care, the consumption of maternal iron tablets during pregnancy, use of contraception, the child’s immunization status, vitamin A supplement capsule, supplementary food intake from the government, weighing of children under five years old, and access to health facilities.

Antenatal care (ANC), as another healthcare sub-variable, involves pregnancy examination by a doctor or trained health worker (first examination during the first trimester of pregnancy, second examination during the second trimester of pregnancy, third and fourth examinations during the third trimester of pregnancy). The data beyond the determined categories were categorized as not performing ANC. Iron tablets are nutritional supplements in the form of tablets/caplets/capsules for boosting iron levels, and they can be obtained from the ANC program or bought during pregnancy. The WHO recommends a daily iron and folic acid supplementation scheme for pregnant women, namely, iron (30–60 mg) as FeSO4 and folic acid (400 g, or 0.4 mg) [28]. It is recommended that pregnant women consume iron tablets every day. However, the Indonesian government, in a regulation announced by the Minister of Health, provides guidelines for a minimum of 90 tablets containing 30–60 mg of iron in the form of ferro sulfate, ferro fumarate, or ferro gluconate, and 0.4 mg of folic acid during pregnancy [29]. The mothers should consume the iron tablets once they have obtained them. The number of tablets consumed according to the mothers’ memory answers was grouped into two categories: <90 tablets (0–89 tablets) and >=90 tablets, in compliance with the mandate of the Ministry of Health program [29]. Contraception is defined as the first modern family planning method that mothers received after giving birth to their last child.

According to the Indonesian Ministry of Health program, basic immunization is considered complete if a child has received one hepatitis B vaccine (HB-0) at birth (or as soon as possible), one dose of Bacillus Calmette–Guerin (BCG) at four weeks of age, three doses of diphtheria, pertussis, and tetanus, (DPT) with hepatitis B (HB) (DPT-HB) at 8, 12, and 16 weeks of age, four doses of the oral polio vaccine (OPV) at 4, 8, 12, and 16 weeks, and one dose of the measles vaccine at age 9 months. The immunization procedures should comply with the immunization guidelines [30]. In the immunization completeness category, it is noted that immunization standards must be obtained at specific age levels. Children aged 6–59 months receive vitamin A supplement capsules from the government according to the standard; children aged 6 months to 11 months receive the capsules once a year; and children aged 12 months receive them twice a year. In the last 12 months, children aged 6–59 months received supplementary food programs from the government such as biscuits, powdered milk, liquid milk, raw food items, and cooked food. Supplementary food from the government was divided into yes and no. Additionally, weighing was an activity for monitoring a child’s growth by measuring their height and weight. This activity can be carried out at Posyandu (the Health and Nutrition Integrated Service Center—a community-based effort) or at a health facility when a child needs care or treatment. Weighing frequency was divided into one or more times per year and never.

Furthermore, numerous questions were used to find out information about access to health facilities at the household level. The principal component analysis (PCA) method was used to analyze three dimensions of information about health facilities, namely: (1) the types of transportation used to visit health facilities; (2) the round-trip time from the home to the health facility; and (3) the round-trip transportation cost to the health facility. The health facility access was categorized as easy (score ≥ mean/median) or difficult (score < mean/median) [21]. Social security is a form of guarantee for the fulfillment of the basic needs of a decent life for each insurance participant and/or their family members. Forms of social security include a veterans’ pension insurance, old age insurance, work accident insurance, death insurance coverage, or severance pay for layoffs. If a household has one of these types of security, it was categorized as having social security. The Program Keluarga Harapan (Family Hope Program) is a conditional social assistance program for poor families. The Social Protection Program, also known internationally as conditional cash transfers (CCT), improves access for poor families, especially pregnant women and children, to take advantage of various health service facilities and educational service facilities available around them. The poverty criteria set by the government form the basis of the acceptance requirements. This program also provides additional assistance for poor household members, namely, pregnant women, early childhood, elementary, junior high, and high school children, the elderly, and people with disabilities, with a maximum of four people in the family. Those considered to be the recipients of the Social Protection Program were households receiving cash transfers. In addition to health facilities, this study also includes the ownership of toilets, divided into two categories: private and joint restrooms.

### 2.6. Statistical Analysis

The data used were sourced from the 2018 Basic Health Research Survey (Riskesdas). Since the samples were collected in stages (multistage), complex sample analysis was performed to conduct the required analysis. To eliminate bias from the population average, complex sampling and a particular data analysis technique with a complex sample design that included weights, clusters, and stratification, were used during sampling. Estimates are taken into account when producing national figures [31]. The distribution of the samples given their features was the result of descriptive analysis. Bivariate analysis using the chi-square test was conducted to determine the distribution of the dependent variables based on the independent variable and its relationship. We used multivariate logistic regression backward stepwise to identify factors associated with stunting. Logistic regression backward stepwise was performed by initially entering all independent variables, then removing them one by one until only significant variables were discovered. The final model kept variables that were significant at the 0.05 level of significance. The results were presented in the form of adjusted odds ratios (OR) and 95% confidence intervals (CI). All analyses were performed using Stata S.E. 15 (Stata Corp, College Station, TX, USA).

## 3. Results

Table 1 shows sample characteristics demonstrating that 768 children 6–59 months of age were located in Bali’s urban areas: 366 of them were boys, and 402 were girls. Of the whole proportion, 19.0% of children in urban areas were stunted. Over 80% of mothers had at least a high school education; delivery at >=20 years to <=35 years; desired the pregnancy; and almost 90% had complete ANC. However, only half of the mothers consumed >=90 iron tablets during pregnancy. Over 70% of children had complete immunization; 61.5% received vitamin A supplements; almost 50% received food from the government; 50.5% lived in extended families/households; and over 90% had been weighed in the past year and had easy access to health facilities. Almost all of the children’s households had improved water sources and their own toilets.

The results of the chi-square test in Table 2 show that the variables of the mother’s education level, antenatal care, the consumption of iron tablets, extended family, and toilet ownership were all associated with stunting in urban children under five years of age (*p* < 0.05). The results of the chi-square test between the mother’s education level and stunting show that mothers with low educational backgrounds have a higher proportion of children with stunting (28.26%) compared to mothers with higher education levels, where the proportion of children with stunting is 16.98%. Mothers who received incomplete antenatal care (26.53%) had a larger prevalence of stunted children than mothers who received complete antenatal care (17.91%). Mothers who consumed <90 iron tablets had a higher proportion of stunted children (22.27%) than mothers who consumed >=90 tablets (16.02%). The proportion of stunted children was higher in non-extended families (22.16%) than in extended families (15.79%). The proportion of children with stunting in households that did not have toilets was 46.15%, much higher than in households that had toilets (18.54%).

Table 3 shows that mothers who did not consume enough iron tablets during pregnancy were associated with a higher risk of stunted children, with an odds ratio of 1.56 (95% confidence interval 1.08–2.24). Regarding the weighting variable, this study showed that children who were never weighed had a lower risk of being stunted, with an odds ratio of 0.40 (95% confidence interval 0.18–0.85). A higher risk of stunting was also associated with the mother’s low education, at 92% (odds ratio of 1.92 (95% confidence interval (1.24–2.97))). The findings of the study also revealed that children who did not have any other adult member in their homes were associated with a higher risk of being stunted, with an odds ratio of 1.55 (95% confidence interval 1.07–2.26).

## 4. Discussion

One of the factors associated with the probability of stunting was the education level of the mother. The findings of several studies conducted in Ethiopia, Rwanda, and Bangladesh are consistent with those of this study [32,33,34]. The likelihood of having a stunted child is inversely related to the mother’s educational level. In Ethiopia, children whose mothers had only completed high school had a higher risk of being stunted, with an odds ratio of 0.10 [33]. A study in Bangladesh showed higher maternal formal education resulted in a 46% reduction in the likelihood of a child being stunted [32]. It has been suggested that a mother’s education has a significant impact on her children’s nutritional status. Mothers who lack education tend to be unaware of the need for good cleanliness and nutrition [33]. This finding highlights the significance of women’s education as an alternative approach to combating childhood stunting and promoting healthy child feeding practices. Higher levels of education could improve the mothers’ knowledge about sanitation practices and healthy behavior to prevent stunting among their children [35]. Educated mothers may have a greater understanding of how to process food, manage food menus, and maintain good food quality and hygiene. In addition to higher education, mothers have to be active and responsive in seeking information about child nutrition from the media and health workers [36].

The findings showed that mothers who did not take at least 90 iron tablets during pregnancy were at risk of delivering children with stunting. The results are in line with the research findings in Ethiopia [37] and Nepal [38], where iron supplementation was associated with maternal and fetal well-being. The regular consumption of iron likely prevents mothers from anemia. A sufficient iron supply in the body will ensure healthy mucosal cell regeneration [39]. Iron supplementation reduces the risk of stunting and low birth weight. The daily consumption of iron tablets throughout pregnancy reduced the likelihood of anemia by 69%, iron deficiency by 66%, and low birth weight by 20% in the intervention group [40]. Additionally, a long-term study conducted in Nepal revealed that children whose mothers took 90 tablets throughout pregnancy had a 22% lower risk of stunting than children whose mothers did not. More specifically, compared to children whose mothers did not take iron tablets, children whose mothers took iron tablets at or before six months of pregnancy had a 16% lower risk of stunting. The risk was relatively reduced by 23% when mothers took 90 tablets at or before six months of pregnancy [38].

The analysis results suggested that the presence of adult family members was associated with a reduced risk of stunting. Our study was consistent with earlier results from Argentina and Indonesia. Studies in West Nusa Tenggara, one of Bali’s surrounding provinces, similarly demonstrated that children who lived in non-extended families were more likely to be stunted than those who were [41]. According to other research from Argentina, children who live in extended families are less likely to experience stunting than children who do not [26]. This finding is intriguing, given that little is known about the beneficial effects of having other family members present in preventing stunting. Families can help with childcare, especially baby care. Stunting is substantially less common in households with three or more dependent people and children under five [27]. The adult presence in the household decreases as the demand for working outside the house increases [42]. Adults who work outside might have economic advantages but less time for childcare, as seen among households in urban areas. Working mothers in the current study made up a significant percentage of the population in urban areas. Evidence in Asia, Africa, and Latin America points to the role of other adults (especially grandmothers), who play a central role as advocates for younger women and as caregivers of women and children on issues of nutrition and health [43]. The purpose of weighing frequency is to monitor growth. Growth monitoring is a type of program that is usually run to reduce stunting in low and middle-income countries (LMICS) [44]. Several studies mention the disadvantage of not attending growth monitoring in stunting. As a study in Ethiopia showed, children who did not attend growth monitoring had a higher likelihood of stunting (adjustedOR = 3.4, 95% Confidence Interval: 1.7–6.9) when compared to children who did [45]. Additionally, in India, stunting was found to be significantly higher among children who did not undergo regular growth monitoring [46]. These findings contradict the results of our study, where coverage is already high but provide a protective effect against stunting. The purpose of monitoring growth can run optimally if several functions are carried out, including teaching mothers and families about how diet and disease can affect children’s growth and providing regular contact with primary health care services when treatment is required [47]. The reasons for the non-optimal role of growth monitoring can be explained according to a UNICEF report where limited counseling was reported in large-scale growth monitoring programs in several countries, including Indonesia, as well as the alleged very limited interpretation of the concept of growth monitoring and neglect to involve caregivers in decision making [47]. The weak interpretation ability of officers on this monitoring concept can lead to a low number of caregivers or mothers who can accurately state their child’s height classification [48].

Some limitations apply to our study. First, it did not investigate other factors, such as maternal complications during pregnancy (hypertension, diabetes, anemia, or inadequate weight gain), children’s nutrition, or children’s morbidity or illness. Second, this study employed a cross-sectional design that could not explain the causal relationship of the stunting variable. Third, the data from several variables were based on the mother’s memory, including the benefit of supplementary food from the government, social security, access to health facilities, use of contraception, the consumption of iron tablets, and the self-reporting of mental conditions. However, the strengths of this study are that it used anthropometric data on children through direct measurements, including vitamin A consumption, weighing, and immunization data, based on the routine observation of children’s health records. Moreover, this study also discussed the condition of household water sources and the ownership of sanitation facilities through direct observation.

## 5. Conclusions

Our study found that, although Bali has a relatively low stunting rate, some disadvantaged residents may face an increased risk of stunting due to some sociodemographic and healthcare factors. Some factors associated with stunting were the absence of other adult family members in the children’s homes, the low level of the mother’s education, and inadequate consumption of iron tablets. This study provides support to the argument that mothers’ education is crucial for managing and preventing stunting. Moreover, in terms of the healthcare industry, pregnant women should be guaranteed to receive iron tablets and encouraged to take sufficient amounts of them. Having other adult family members at the children’s home could help reduce the likelihood of stunting among children, and thus this social factor should be considered when designing stunting interventions. Health and community stakeholders have to work together to accomplish these goals using the local wisdom available in communities.

## Figures and Tables

**Table 1:** Descriptive characteristics of children aged 6–59 months in Bali Province, 2018.

Characteristics	*n*	%
Stunting		
No	622	81.0
Yes	146	19.0
Child’s characteristics		
Child’s sex		
Boy	366	47.7
Girl	402	52.3
Child’s age group		
<24 months	247	32.2
>=24 months	521	67.8
Child’s immunization status		
Complete	582	75.9
Incomplete	285	24.1
Receiving vitamin A		
Yes	472	61.5
No	296	38.5
Acceptance of supplementary food from the government		
Yes	371	48.3
No	397	51.7
Weighing frequency		
One or more times per year	697	90.76
Never	71	9.24
Mother’s characteristics		
Mother’s level of education		
Senior high school and above	630	82.0
Junior high school and below	138	18.0
Mother’s employment status		
Working	505	65.8
Not working	263	34.2
Mother’s emotional mental disorder		
No	715	93.1
Yes	53	6.9
Mother’s age at delivery		
>=20 to <=35 completed years	636	82.8
<20 or >35 completed years	132	17.2
Desired pregnancy		
Desired	621	80.9
Undesired	147	19.1
ANC		
Complete	670	87.2
Incomplete	98	12.8
Iron tablets		
>=90 tablets	412	53.6
<90 tablets	356	46.4
Use of modern contraception		
Yes	505	66.1
No	259	33.9
Household’s characteristics		
Economic status		
Three top wealth quintiles	388	50.5
Bottom two wealth quintiles	380	49.5
Extended family		
Yes	380	49.5
No	388	50.5
Access to health facilities		
Easy	713	92.8
Difficult	55	7.2
Water Sources		
Improved	761	99.1
Not improved	7	0.9
Social Security		
Yes	136	17.7
No	632	82.3
Receipt of government’s social protection program		
Yes	63	8.2
No	705	91.8
Ownership of toilet		
Owning	755	98.3
Not owning	13	1.7
Total	768	100.0

**Table 2:** Chi-square analysis of the sociodemographic and healthcare factors with stunting status among children aged 6–59 months in Bali Province, 2018.

Characteristics	No Stunting		Stunting		*p*-Value
	*n*	%	*n*	%	
Child’s characteristics					
Child’s sex					0.304
Boy	302	82.51	64	17.49	
Girl	320	79.60	82	20.40	
Child’s age group					
<24 months	202	81.78	45	18.22	0.700
>=24 months	420	80.61	101	19.39	
Child’s immunization status					0.304
Complete	476	81.79	106	18.21	
Incomplete	145	78.38	40	21.62	
Receiving vitamin A					0.118
Yes	374	79.24	98	20.76	
No	248	83.78	48	16.22	
Acceptance of supplementary food from the government					0.081
Yes	291	78.44	80	21.6	
No	331	83.21	66	16.62	
Weighing frequency					0.081
One or more times per year	559	80.20	138	19.80	
Never	63	88.73	8	11.27	
Mother’s characteristics					
Mother’s education level					0.002*
Senior high school and above	523	83.02	107	16.98	
Junior high school and below	99	71.74	39	28.26	
Mother’s employment status					0.332
Working	414	81.98	91	18.02	
Not working	208	79.09	55	20.91	
Mother’s emotional mental disorder					0.289
No	582	81.40	133	18.60	
Yes	40	75.47	13	24.53	
Mother’s age at delivery					0.150
>=20 to <=35 completed years	521	81.92	115	18.08	
<20 or >35 completed years	101	76.52	31	23.48	
Desired pregnancy					0.825
Desired	502	80.84	119	19.16	
Undesired	120	81.63	27	18.37	
ANC					0.042 *
Complete	550	82.09	120	17.91	
Incomplete	72	73.47	26	26.53	
Iron tablets					0.023 *
>=90 tablets	346	83.98	66	16.02	
<90 tablets	276	77.53	80	22.27	
Use of contraception					0.497
Yes	405	80.20	100	19.80	
No	213	82.24	46	17.76	
Household’s characteristics					
Economic status					0.489
Three top wealth quintiles	318	81.96	70	18.04	
Bottom two wealth quintiles	304	80.00	76	20.00	
Extended family					0.024 *
Yes	320	84.21	60	15.79	
No	302	77.84	94	22.16	
Access to health facilities					0.871
Easy	577	80.93	136	19.07	
Difficult	45	81.82	10	18.18	
Water sources					0.106
Improved	618	81.21	143	18.79	
Not improved	4	57.14	3	42.86	
Social Security					0.158
Yes	116	85.29	20	14.71	
No	506	80.06	126	19.94	
Receipt of Government’s social protection program					0.178
Yes	47	74.60	16	25.40	
No	575	81.56	130	18.44	
Ownership of toilet					0.012 *
Owning	615	81.46	140	18.54	
Not owning	7	53.85	6	46.15	

Note: * *p* < 0.050.

**Table 3:** Multivariate logistic regression analysis for association of stunting status with sociodemographic and healthcare factors among children aged 6–59 months in Bali Province, 2018.

Variable	adjustedOR	95% Confidence Interval	*p*
Mother’s level of education			
Senior high school and above	1		
Junior high school and below	1.92	(1.24–2.97)	0.003 *
Iron tablets			
>=90 tablets	1		
<90 tablets	1.56	(1.08–2.24)	0.018 *
Extended family			
Yes	1		
No	1.55	(1.07–2.26)	0.022 *
Weighing frequency			
One or more times per year	1		
Never	0.40	(0.18–0.85)	0.018 *

Note: * *p* < 0.050.

## Data Availability

The data presented in this study are available upon request to the corresponding author. The data are not publicly available due to restrictions concerning privacy and ethical reasons.
